# Low-phosphate-selected *Auxenochlorella protothecoides* redirects phosphate to essential pathways while producing more biomass

**DOI:** 10.1371/journal.pone.0198953

**Published:** 2018-06-19

**Authors:** Sang-Hyuck Park, John Kyndt, Kapeel Chougule, Jeong-Jin Park, Judith K. Brown

**Affiliations:** 1 Department of Biology, Colorado State University, Pueblo, Colorado, United States of America; 2 College of Science and Technology, Bellevue University, Bellevue, Nebraska United States of America; 3 Arizona Genomics Institute, The University of Arizona, Tucson, Arizona, United States of America; 4 Biomolecular Analysis Facility, University of Virginia, Charlottesville, Virginia, United States of America; 5 School of Plant Sciences, The University of Arizona, Tucson, Arizona, United States of America; International Centre for Genetic Engineering and Biotechnology, INDIA

## Abstract

Despite the capacity to accumulate ~70% w/w of lipids, commercially produced unicellular green alga *A*. *protothecoides* may become compromised due to the high cost of phosphate fertilizers. To address this limitation *A*. *protothecoides* was selected for adaptation to conditions of 100× and 5× lower phosphate and peptone, respectively, compared to ‘wild-type media’. The *A*. *protothecoides* showed initial signs of adaptation by 45–50 days, and steady state growth at ~100 days. The low phosphate (P)-adapted strain produced up to ~30% greater biomass, while total lipids (~10% w/w) remained about the same, compared to the wild-type strain. Metabolomic analyses indicated that the low P-adapted produced 3.3-fold more saturated palmitic acid (16:0) and 2.2-fold less linolenic acid (18:3), compared to the wild-type strain, resulting in an ~11% increase in caloric value, from 19.5kJ/g for the wild-type strain to 21.6kJ/g for the low P-adapted strain, due to the amounts and composition of certain saturated fatty acids, compared to the wild type strain. Biochemical changes in *A*. *protothecoides* adapted to lower phosphate conditions were assessed by comparative RNA-Seq analysis, which yielded 27,279 transcripts. Among them, 2,667 and 15 genes were significantly down- and up-regulated, at >999-fold and >3-fold (adjusted *p*-value <0.1), respectively. The expression of genes encoding proteins involved in cellular processes such as division, growth, and membrane biosynthesis, showed a trend toward down-regulation. At the genomic level, synonymous SNPs and Indels were observed primarily in coding regions, with the 40S ribosomal subunit gene harboring substantial SNPs. Overall, the adapted strain out-performed the wild-type strain by prioritizing the use of its limited phosphate supply for essential biological processes. The low P-adapted *A*. *protothecoides* is expected to be more economical to grow over the wild-type strain, based on overall greater productivity and caloric content, while importantly, also requiring 100-fold less phosphate.

## Introduction

Biofuels produced by microalgae have great potential to supplement traditional, petroleum-based transportation fuels without disrupting food production or causing deleterious environmental effects. Algae can produce one hundred times more oil per surface area, or greater, than traditional oil crops, and constitutes a feedstock for biodiesel, jet fuel, and ethanol [1–3]. Also, oils produced from microalgae can be converted to drop-in fuels. Even so, a major challenge to producing microalgal feedstocks is the rising cost of phosphate fertilizer, potentially becoming prohibitively expensive and so, not cost effective. Algal performance e.g. growth rate and triacylglycerol (TAG) production depends upon phosphate availability, which is essential for cellular processes including energy production, membrane biosynthesis, photosynthesis, protein synthesis, and signal transduction, [4, 5]. Limited phosphate availability can negatively affect chlorophyll content, growth rate, protein synthesis, and the accumulation of β-carotene, glycerol, and total soluble carbohydrates [4–6]. Another consequence of limited availability of phosphate and nitrogen is known to result in accumulation of lipids in algal cells, which is stored and used for energy when conditions are again favorable for growth [7]. Specifically, for *A*. *protothecoides*, a nitrogen-deficient/phosphate-repletion cultivation strategy was reported to result in increases in lipid yields, by as much as 55% (Li et al. 2014).

*Auxenochlorella protothecoides (Krüger) Kalina & Puncochárová, 1987* is a unicellular microalga with the capacity to accumulate high levels of triacylglycerols (TAGs) through heterotrophic cultivation in bioreactors [8]. However, it requires substantial phosphate inputs during the scale-up phase of production. Phosphate is expected to become limited as a natural resource in the very near future, and it has become expensive even for current uses. Consequently, the scale-up phase in commercial production systems will increasingly rely on the cost-effective use of phosphate fertilizers [9]. Thus, the potential accessibility of phosphate-efficient algal strains offers some promise for reducing the amount of phosphate required for scale-up, which could have a direct effect on the feasibility of cost-effective algal biofuel production.

Maintaining algal cultures under suboptimal conditions for temporary periods of time provides a practical approach for the selection of new genetic variants from among a diverse population. Using an ‘adaptive evolution’ strategy to select new biological variants or ‘strains’ has been commonly applied to direct and/or step-up the evolution of many organism already used for commercial purposes e.g., *Escherichia coli* [10], yeast [11], and microalgae [12]), and includes diverse products used in medicines, nutritional supplements, food, beverages, and TAGs, among others.

Naturally-occurring mutations are the primary sources of genetic variation that can result in increased organismal fitness in response to environmental change. Most spontaneous mutations are considered likely to be deleterious, whereas, others, often considered to be rare, can be beneficial to an organism by enabling adaptation to adverse conditions, often imposed, for example, by nutritional- or temperature-induced stresses. In a recent study, when subjected to adverse conditions, *Chlamydomonas reinhardtii* was found to rapidly accumulate mutations that resulted in altered fitness [13].

Differential gene expression analysis has become commonplace among new analytical tools to inform the efficiency of biochemical pathways that drive cellular and molecular processes, including for algae [9, 14]. When combined with genome-level characterization of exons, introns, and the number of exon isoforms and splice sites, the collective data sets offer great insights into functional predictions, and predicted sites of post-transcriptional modification [15].

Microalgae produce diverse lipid compounds whose compositional profiles vary, depending on cultivation conditions such as pH and temperature, and nutrient deprivation that leads to starvation [16, 17]. The analysis of fatty acids and other lipid compositions based on mass spectrometry analyses of algal cultures at different growth or ‘cultivation’ stages have been used to monitor cultures for nutrient limitation. As essential nutrients, lower than optimal compositions of phosphate or nitrogen in culture media, can dramatically affect fatty acid and lipid composition and algal growth rate e.g. biomass. And importantly, fatty acid composition significantly influences fuel properties of synthesized biodiesel [16].

In this study, a low P-variant of *A*. *protothecoides* was selected by subjecting it to ‘adaptive evolution’, using a chemostat cultivation system. During the selection process, challenges involved maintaining cultures at exponential growth stage and free of microbial contamination. Throughout the adaptation process, cells were periodically collected and subjected to RNA-Seq analysis of expression profiles of the presumed, evolving populations.

Gene expression analysis was used to inform transcript abundance at 2-wk time intervals throughout the adaptation process. Templated mapping of the RNA-Seq transcripts to the reference genome *A*. *protothecoides* sequence was performed to document changes associated with selected genes of interest, by assessing single nucleotide (nt) polymorphisms (SNPs) and insertions and deletions (Indels) [18, 19]. For selected time points, the composition of fatty acids and lipids were analyzed, and calorific values were determined to predict how compositional changes affected the overall energy production.

The chemostat cultivation system proved to serve as an effective culture system for selecting a viable, low P-adapted algal strain. This *A*. *protothecoides* low P-adapted strain selected by classical ‘adaptive evolution’, is expected to have great potential for large-scale algal cultivation under low phosphate conditions, by enabling the production of greater than wild-type algal biomass per phosphate input and at a lower cost, thus, providing potentially major economic advantages to biodiesel markets.

## Materials and methods

### Algal selection in proteose media

Proteose medium was prepared by the addition of 10ml/L from the stock solutions: 3.7g/L MgSO_4_, 25g/L NaNO_3_, 2.5g/L CaCl_2_·2H_2_O, 17.5g/L KH_2_PO_4_, 7.5g/L K_2_HPO_4_, and 2.5g/L NaCl in 1.0 g/L of proteose-peptone solution. The pH was adjusted to 7.0 using 1M NaOH and sterilized by autoclaving. The low phosphate/ low peptone media was prepared using the traditional standard proteose media, except KH_2_PO_4_ and K_2_HPO_4_ were decreased 100× fold (0.175 g/L and 0.075 g/L respectively), and proteose-peptone media was decreased 5× fold (0.05 g/L).

### Algal adaptation under low phosphate conditions

The culture of *A*. *protothecoides* was obtained from the Culture Collection of Algae (https://utex.org), located at the University of Texas at Austin (UTEX #25), Austin, TX. Batch cultivation of *A*. *protothecoides* was performed in either 250 ml or 1 liter erlenmeyer flasks with a culture volume of 50 ml and 250 ml respectively. Batch cultures were continuously bubbled with a 5% CO_2_/95% air mixture during growth at a temperature of 28°C. Batch cultures were started with a 5% incolum from a stationary phase culture. For the continuous chemostat cultivation, late-exponential phase *A*. *protothecoides* (800 ml) pre-culture was inoculated into a 14 liter fermenter containing 1/100 phosphate and 1/5 peptone proteose media. *A*. *protothecoides* was subjected to prolonged growth (> 4 months) in a chemostat with constant selection under low phosphate to stimulate adaptation to the altered environmental conditions. The chemostat air, pH and flow rates were monitored and controlled. The temperature was held at 25–28°C. In a chemostat setting a certain amount of the culture is harvested and constantly replaced with an equivalent of fresh media [20]. This was performed while cultures were in exponential phase so that there is a minimal lag phase during re-growth of the culture. The culture will grow with a specific reproductive rate: μ = μmax (s/ (Ks+s)) where μmax = max rate when all nutrients are present, s = concentration of limiting supply, and Ks = concentration of limiting nutrient at half growth rate. Low phosphate media were added with a dilution rate D = f/v where f = rate of fresh media added, v = vessel volume, and D = dilution rate. By diluting the culture with a media that has a limiting amount of one essential nutrients, in this scenario, the phosphate/peptone, the growth rate (μ) was controlled in the chemostat, based on the dilution rate D of the limiting nutrient, so that μ = D. To estimate the final biomass yield estimations, the OD values from the growth curves were converted to g/L ash-free dry weight (AFDW), based on a calibration curve previously determined for multiple growth curves of the same wild type *A*. *protothecoides* isolate cells grown in open pond and in closed indoor photobioreactor experiments.

### Lipid extraction and analysis

One liter of culture was centrifuged under 4500× g for 30 min. The supernatant was discarded and the algae pellet stored at -80°C. The frozen samples were dried overnight in a vacuum desiccator, with sodium hydroxide. The dried algal samples were ground into a fine powder, placed in an extraction thimble, and weighed. The thimble was placed in a glass beaker and chlorophorm was added to cover the powder. The algal suspension was sonicated on ice for 5 min (output 4 on an Ultrasonic Inc., Cell Disruptor model W-220F with a microtip). After sonication, the thimble and chlorophorm were placed in a Soxhlet extractor and lipids were extracted at 66–70°C for 4–6 hrs, and the excess chloroform was removed by distillation. The concentrated lipids and residual chloroform was transferred to a pre-weighed vial and dried overnight to remove chloroform. The vial was weighed, and the lipid content by dry weight was determined as a percentage of the dry biomass weight: dry weight of lipids/ dry weight of algae × 100%.

### Flow cytometry

The wild-type and low P-adapted strain, both grown under low PO_4_/low peptone conditions, were analyzed by flow cytometry (FC) on an Attune Flow cytometer (Life Technologies, NY, Grand Island) to compare the cell population size (number) (using intrinsic chlorophyl fluorescence) and compare lipid composition (using BODIPY 505/515). Chlorophyll fluorescence was used for detection (> 640 nm) of all cells. FC experiments were carried out using BODIPY 505/515 staining to compare lipid quantities between samples. BODIPY fluorescence was detected using a 530 nm band pass filter.

### Fatty acids analysis by GC-FID

Gas chromatographic analysis of fatty acids was carried out using a Hewlett-Packard HP 5890 gas chromatograph equipped with a split–splitless injector and flame ionization detection (FID) system (Hewlett-Packard, Palo Alto, CA), at 260°C, using helium as the carrier gas. A 1 μl sample volume was injected using a split-mode, at 1:40. The Econo-Cap capillary column (30 m length, 0.25 mm I.D., 0.25 μm film thickness) (Alltech, Deerfield, IL) used for the analysis was programmed over a range of 150°C (3 min-hold) to 240°C (5 min-hold) at 10°C/min. The resultant peaks were quantified based on peak area using a standard reference library.

### Lipid profiling by LC-MS

Chromatographic separation was performed using an AQUITY-UPLC system (Waters, Manchester, UK) equipped with a BEH-C18-column, 2.1 × 150 mm, 1.7 μm (Waters), using a binary gradient of two solvents, A: water/methanol (1/1, v/v) and B: 2-propanol, made in phosphoric acid (8 μM), ammonium acetate (10 mM) and formic acid (0.1 vol %). The initial linear gradient consisted of solvent B at 45%, followed by an increase 90% within 30 min, and final increase to 100% for 10 min, at 50°C. Starting conditions were established at 1 min and the column was re-equilibrated for 7 min, resulting in a 50 min LC run time. A Synapt G2-s mass spectrometer (Waters) equipped with an ESI source was used for the analysis. The source parameters were: capillary temperature 110°C, desolvatization temperature: 250°C, nitrogen as nebulizer gas. The capillary voltage was 3.0 kV and 2.7 kV in the positive and negative ionization modes, respectively. For MSE, two alternating scan modes were defined for the MS setup step. The first scan mode was carried out at the full scan mode (mass range: m/z 50–1800; scan time: 1s; data collection: centroid), followed by a scan mode (mass range: m/z 50–1800; scan time: 1s; data collection: centroid) using a collision energy ramp (25–45 V) to fragment ions. Both scan modes showed a resolution of approximately 9000 (FWHM). For the MS/MS experiments, the same settimgs for scan time and collision energy were used. Data acquisition was carried out using MassLynx 4.1 software (Waters, Milford, MA). Lipid analysis was carried out using Progenesis QI software (Waters), and specific lipids were identified based on searches carried out using LipidsMaps (http://www.lipidmaps.org/).

### Calorimetry

The wild-type and low P-adapted *A*. *protothecoides* samples were oven-dried at 55°C for 24 hrs. The total energy/per gram was determined using by bomb calorimetry with a Parr 6725 semi-micro calorimeter, 1109A semi-micro oxygen bomb, and 6772 thermometer (Parr Instrument Co., Moline, IL), using the calorimeter model standard operating procedures (http://faculty.ycp.edu/~jforesma/educ/pchem/parr-calor.pdf). The calorimetry energy equivalent factor was determined using benzoic acid standards (Sigma-Aldrich, St. Louis, MO). To calculate the percentage increase of calorific value, the following equation is used: % calorific value increase = (calorific value of low P-adapted–calorific value of wild-type strain) / calorific value of wild-type strain × 100.

### SNP/Indel and RNA-Seq analysis

To identify single gene mutations, e.g., SNP and Indel, in the low P-adapted *A*. *protothecoides*, The RNA-Seq reads produced for the different time periods (2, 6, 10, >14, and >22 wks) were assembled using the annotated wild-type *A*. *protothecoides* genome as a reference by DNASTAR SeqMan NGen software (DNASTAR, Inc., Madison, WI). The SNP and Indel calling was achieved by mapping reads directly to the annotated reference genome. The identified SNP/Indels were filtered at Q-value > 60 and >100× coverage.

For RNA-Seq analysis, the progenitor *A*. *protothecoides* was grown for adaptation under 100× lower phosphate and 5× lower peptone proteose growth media for >22 wks. To carry out a time-course analysis of gene expression over the adaptation period, total RNA was isolated from algal cells sampled at two-wk intervals. One hundred ml of algal suspension cultures were harvested at the exponential growth phase for the 2, 6, 10, >14, and >22 wks generation with three replicates except for the two low P-adapted strains (>14 and >22 wks) that has only one sample harvested. The algal cells were harvested by centrifugation at 3,600 ×g for 8 min at 4°C. The pellet was washed twice in autoclaved, double-distilled water (ddH_2_0) and total RNA was isolated and purified using a RNAse Mini Kit (QIAGEN Inc., Valencia, CA). The RNA was quantified using a Nanodrop 2000 (Thermo Scientific Inc. Wilmington, DE), and messenger RNA (mRNA) was selected from total RNA by using the FastTrack MAG beads. The mRNA preparation, including cDNA library formation, was performed using the Illumina TruSeq RNA Sample Preparation Kit v2 (Illumina, Inc. San Diego, CA), according to the manufacturer’s protocol.

The trasncripts were annotated using the MAKER annotation pipeline [21]. The MAKER pipeline carries out repeat-masking of contigs using gene prediction tools. The Augustus and SNAP algorithms were used to predict gene identifications, and to align RNAs/ESTs to the reference genome determined by the Los Almos National Laboratory for the National Alliance for Advanced Fuels and Biofuels Project [22], yielding synthesized annotated gene models as outputs.

The Illumina RNA-Seq raw sequences were deposited in the NCBI GenBank database as the biosamples: SAMN08322802, SAMN08322807-25 under bioproject PRJNA428835. The assembled RNA-Seq was archived in the CyVerse Data Commons (DOI 10.7946/P2VH11). The RNA-Seq reads were normalized by RPKM (Reads Per Kilobase of transcript per Million mapped reads). The RNA-Seq sequences were assembled by mapping reads directly to the annotated reference transcripts using the DNASTAR SeqMan NGen software (DNASTAR, Inc., Madison, WI). Differential gene expression levels were quantified using Fisher’s Exact Test Signal Search available in the DNASTAR ArrayStar software package (DNASTAR, Inc., Madison, WI). The False Discovery Rate (FDR) for the multiple testing was corrected using the Benjamini Hochberg algorithm, with adjusted *p*-value of 0.1 as a cutoff.

## Results

### Sub-optimal phosphate growth conditions

**Fig 1:**
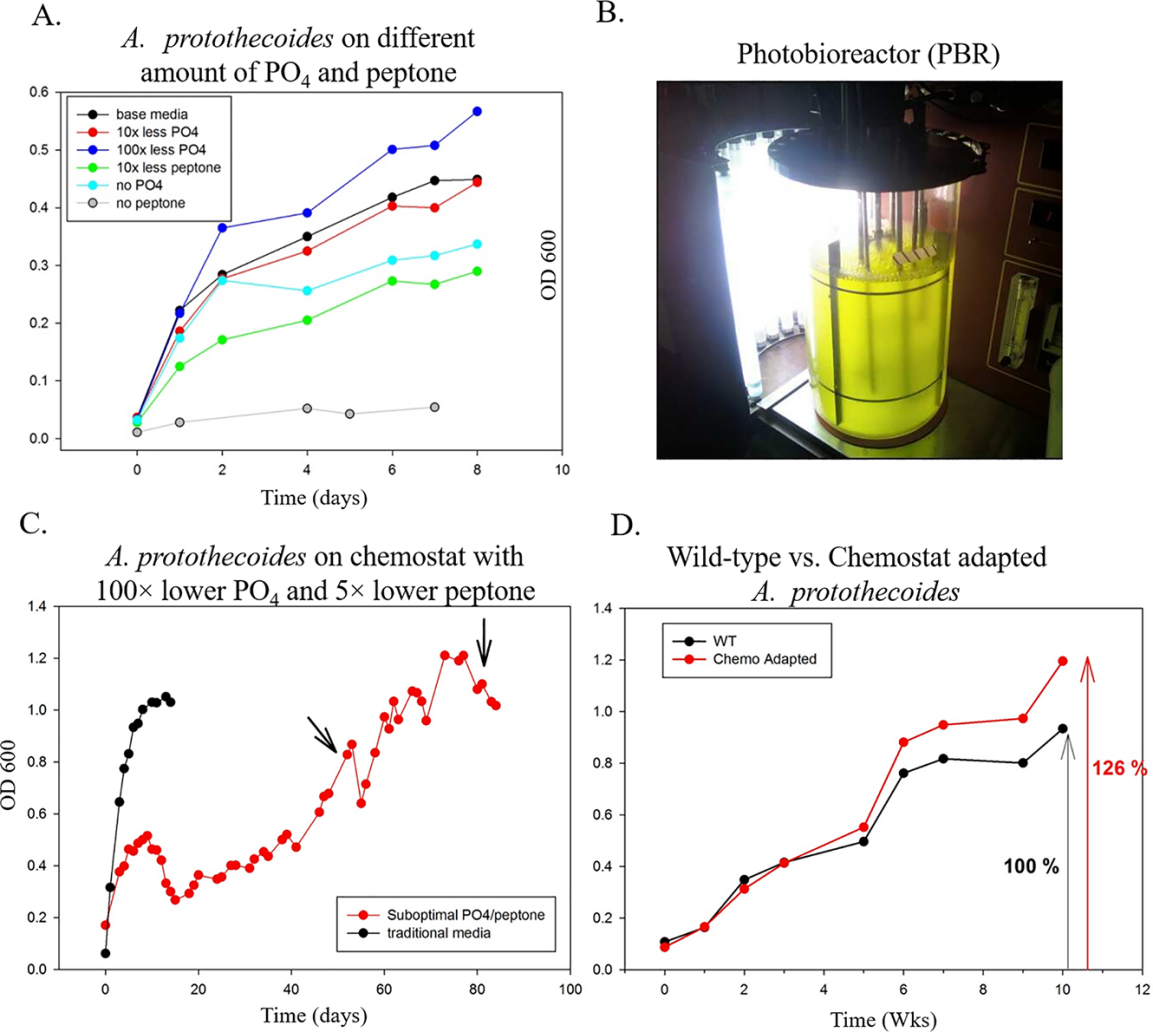
*A*. *protothecoides* growth performance under a suboptimal condition (100× lower phosphate and 5× lower peptone). **A.** Limits of growth of *A*. *protothecoides* in varying amounts of phosphate and peptone. **B.** Chemostat growth in traditional proteose and suboptimal phosphate and peptone media. **C.** Side-by-side growth comparison of *A*. *protothecoides* on traditional- and suboptimal PO_4_/peptone media. **D**. Side-by-side growth comparison between wild-type and low P-adapted *A*. *protothecoides* cultures under suboptimal PO_4_/peptone media.

*A*. *protothecoides* was grown in proteose peptone media with varying amount of peptone and/or phosphate in 250 ml batch cultures. Inorganic phosphate concentrations analyzed varied from 1/10, 1/100 to 0. Peptone concentrations were lowered from 1/10 of the original media to zero peptone added. The resulting growth curves are shown in Fig 1A. Preliminary results showed that initial growth rate and biomass yield were only minimally affected by the 100× reduced inorganic PO_4_, implying that the base media contains excess of inorganic PO_4_ with respect to the amount required for growth. The PO_4_ in the media was not limiting, and interestingly, the 100× lower PO_4_ was found to increase the overall biomass yield by ~15% (Fig 1A). Thus, the reduction in the amount of proteose peptone had a much more significant effect on growth and so it was necessary to maintain this constituent at a concentration of at least 0.5 g/l, which is 2× lower than for traditional media to obtain a growing culture. A condition of 10× lower peptone combined with 100× lower PO_4_ did not result in significant growth (data not shown). Based on these observations the target for suboptimal growth was established at 5× lower peptone and 100× lower PO_4,_ compared to traditional proteose peptone media.

### Adaptation of *A*. *protothecoides* to low phosphate conditions

*A*. *protothecoides* was grown under the 100× lower PO_4_ and 5× lower proteose peptone media suboptimal conditions in a chemostat setting (Fig 1B). Initially a baseline growth was established with the normal proteose peptone media in the chemostat (black curve in Fig 1C), reaching an OD_600_ of ~1.1. When the culture was grown under suboptimal phosphate/peptone conditions the initial OD_600_ was only about 0.45, which is consistent with our growth limit studies performed in batch. Potential signs of adaptation were observed after ~45 days in the chemostat with low PO_4_/peptone growth. With the culture constantly being kept under suboptimal PO_4_/peptone conditions, the optical density (OD) of the culture in the chemostat started to increase and stabilized at a value about three times higher than before the adaptation (~0.45 vs 1.2; Fig 1C). Samples were taken at the peak OD values and used for further growth analysis (rate and biomass yield) and for lipid extraction. DNA was also extracted from the chemostat culture to perform species identification to exclude contamination. Previously designed 18S rRNA gene primers were shown to work to identify algae from lab cultures and cultures grown in open raceway for extended time. This 18S rRNA primer detection approach was used to assure that the culture after adaptation in the chemostat was the same as the initial *A*. *protothecoides*. Also, the 16S rRNA sequence analysis revealed no difference in relative bacterial contamination during prolonged chemostat growth.

After the chemostat adaptation, follow-up experiments were designed in batch cultivation (1-liter cultures) to compare the growth performance of the wild-type and adapted strain. These side by side comparisons of chemostat-adapted strain compared to wild-type generated at least 25% more biomass under a similarly low PO_4_ concentration based on optical density measurements (Fig 1D). When comparing biomass yields as AFDW, this corresponds to an average increase in biomass yield of 30%, from 0.230 g/L for the wild-type strain to 0.293 g/L for the low P- adapted strain.

Lipid extractions were performed on algal samples from the chemostat after the initial indication of adaptation, consisting of 1-liter volumes, collected at an O.D. of ~0.85. The total lipids were extracted with a Soxhlet column with chloroform. Lipid yield was 11.6%, which was comparable to the pre-chemostat values observed of ~10%.

### Flow cytometric analysis of wild-type and low phosphate-adapted *A*. *protothecoides*

Flow cytometry experiments were performed on wild-type and the low P-adapted population to determine potential differences in cell composition and lipid content. Both wild-type and selected population were grown under low PO_4_/low peptone conditions for these experiments. Flow cytometry was performed on an Attune Flow cytometer (Life Technologies, NY, Grand Island). Chlorophyll fluorescence was detection using a long pass filter > 640 nm. Fluorescence intensity was high for both samples when illuminated with violet light. Both wild-type and adapted samples are shown as density plots (left) and histogram (right). Although microscopic and 18S rRNA gene analyses did not show any signs of contaminants or mixed population, a secondary population with higher fluorescence was present in both flow cytometry samples. It has been observed that *A*. *protothecoides* forms clusters of cells as the culture ages and it is therefore highly likely that this secondary population represents the same aging cells that are detectable as a separate population by flow cytometry [23].

**Fig 2:**
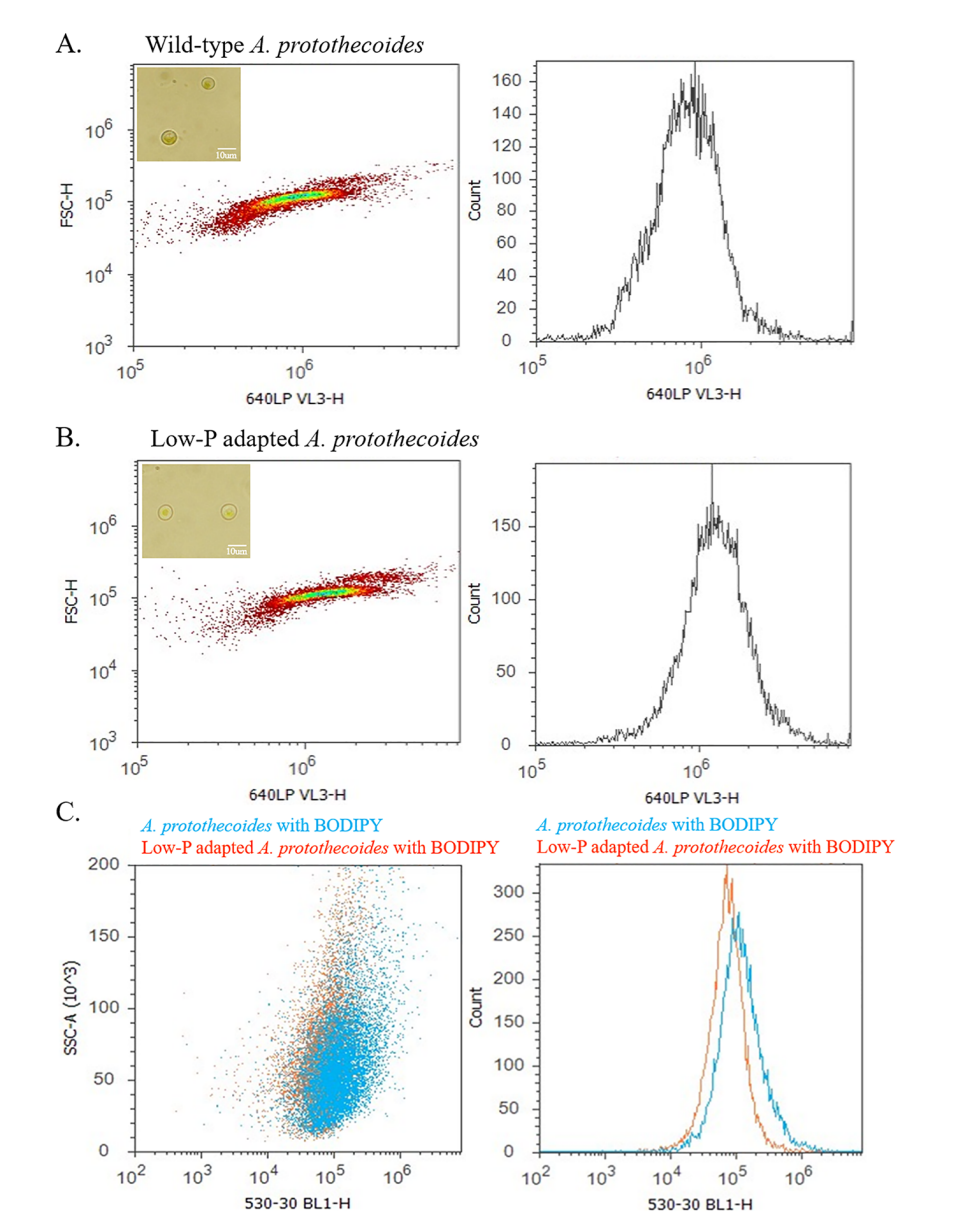
Flow cytometric analysis of wild-type and low P-adapted *A*. *protothecoides*. Density plots and corresponding histograms of wild-type (**A**) and low P-adapted (**B**) were shown for the chlorophyll-based fluorescence. **C.** Density plot and histogram of flow cytometric measurement were based on BODIPY fluorescence.

The flow cytometry experiments were also performed using BODIPY 505/515 stain, to compare lipid quantities in both samples. In this case, the BODIPY fluorescence was detected using a band pass filter at 530 nm. When overlaying the resulting BODIPY staining for wild-type (Fig 2A) and selected population (Fig 2B) lipid content seemed comparable (Fig 2C). This is consistent with previously reported yield of 10 and 11% lipid content, extracted from 1-liter cultures of both samples. A small shift was observed in wild-type cells which indicates slightly more fluorescence per cell, on average about 35% more. Although this percentage might seem significant, this would only represent a minimal variation in the overall extractable lipid yield (~ 3%).

**Fig 3:**
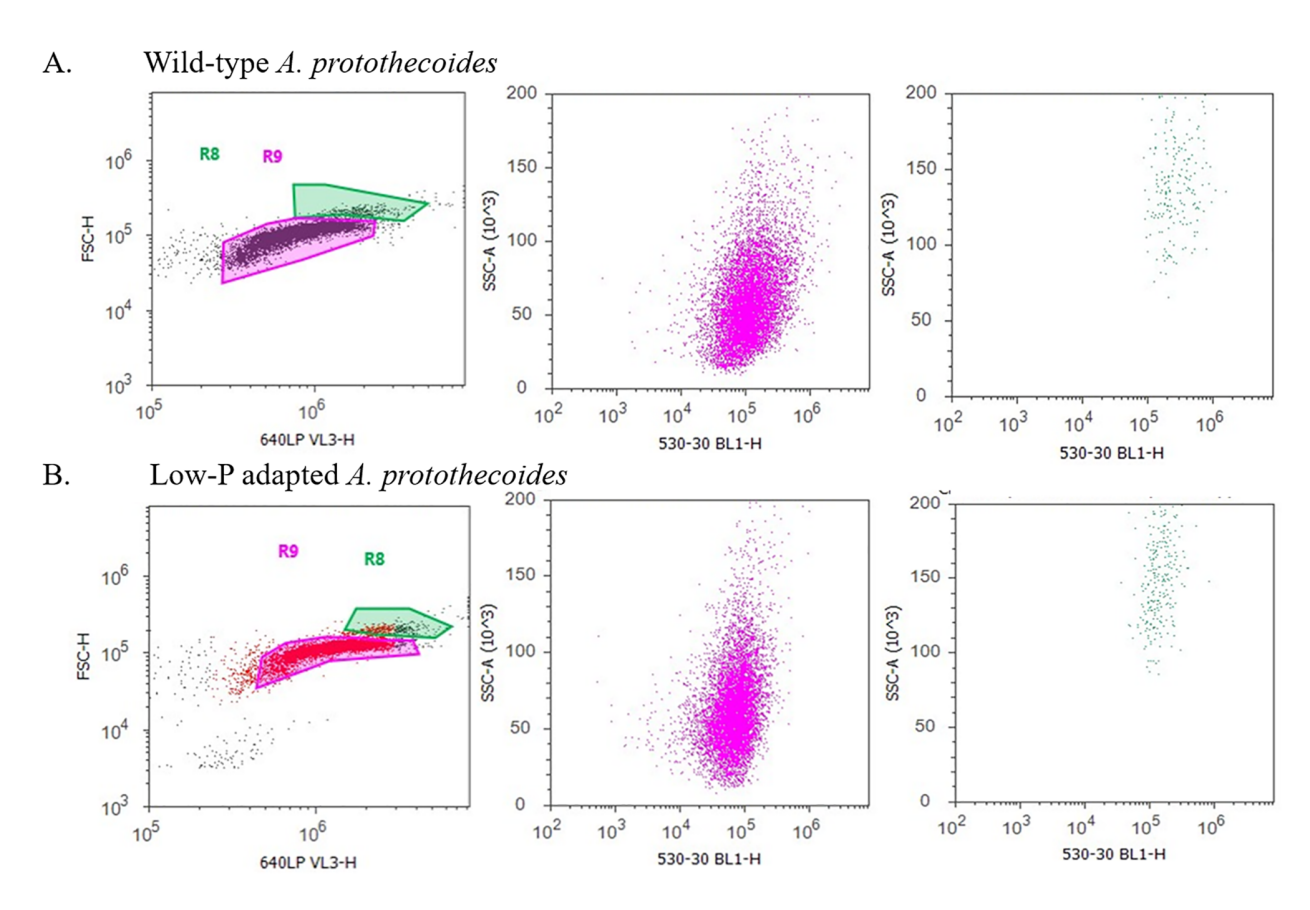
A. Flow cytometry analyses on BODIPY stained wild-type *A*. *protothecoides* (**A**) and low P-adapted strain (**B**). Two strains appearing to represent two separate populations; primary (R9; red) and secondary (R8; green).

Comparison of the lipid content (BODIPY fluorescence) in the wild-type primary and secondary populations, R9 (red) and R8 (green) indicated that the R8 cells had significantly higher BODIPY fluorescence than the R9 cells (Fig 3A), with approximately twice the mean fluorescence value. The same was observed with suboptimally adapted *A*. *protothecoides* BODIPY fluorescence (Fig 3B). This observation suggested that the secondary population represented clusters of *A*. *protothecoides* cells at the onset of senescence, and that as would be expected, their lipid accumulation per cell was greater than for cells in log or stationary growth phases.

### RNA-Seq analysis of SNPs and Indels

To predict the possible genetic mechanism(s) used by *A*. *protothecoides* to adapt to suboptimal phosphate conditions, we investigated the hypothesis that single gene mutations (e.g., SNP, Indel) evident at the genome level in either coding and/or non-coding regions, could compensate for adaptation to the lower phosphate conditions. To scan for mutations, the genome sequence, previously determined using the Illumina Next Generation Sequencing (NGS) platform (HiSeq2000), was scanned for genomic mutations associated with each adaptation step.

**Fig 4:**
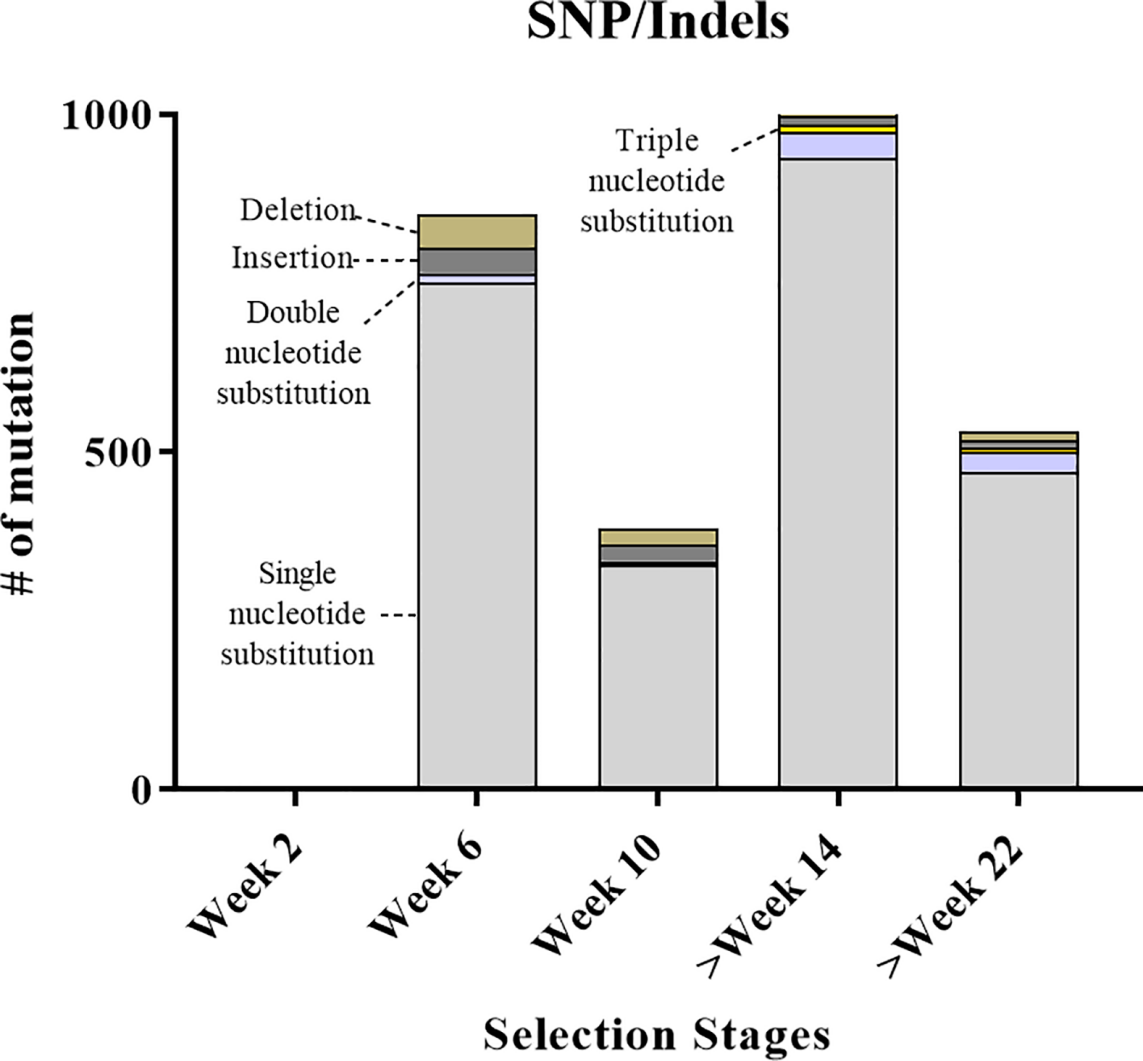
Genomic variants, e.g. SNP/Indels, identified during selection process in low phosphate media. Three types of nucleotide substitutions (single, double, and triple), insertional-, and deletional mutations were indicated in the stacked bar.

Genomic variants (e.g., SNPs/Indels) were identified from samples collected from the different time periods of adaptation (wk 2, 6, 10, >14, and >22), and a number and several types of SNPs/Indels were detected (Fig 4). Transcripts with SNPs/Indels embedded are shown in Table 1. The wk 2 transcripts showed no discernable accumulation of variants, using the parameters Q-value 60 with 100× coverage, which therefore, were set as the default parameters for further analysis. Genetic variants began to accumulate at wk 6, for which 853 SNP/Indels were observed. At wk 10, 388 SNP/Indels were identified, with the number increasing to 1,011 SNP/Indels by wk >14. After 14 wks, *A*. *protothecoides* exhibited 3× higher OD values, compared to the starting population. By >22 wks, the low P-adapted strain had 533 SNPs/Indels, compared to annotated reference genome. Although the genome-wide distribution of genetic variants was not tabulated, SNP/Indel patterns differed by time of sampling, suggesting that genetically variable populations evolved over the course of the adaptation under low phosphate conditions.

**Table 1:** List of SNPs/Indels identified for each selection process, attributed to selection under low phosphate conditions. Each transcript was found to harbor single or multiple SNPs in the coding region.

	Transcripts with SNP/Indels	Ref. Position	SNP/ Indel	Coverage depth (×)	Q-value	SNP %
**Week 6**	**Cpro00002392**—*Auxenochlorella protothecoides* strain 0710 sucrose phosphate synthase	431	T>C	51	60	100%
	**Cpro00002508**—*Chlorella protothecoides* mRNA for putative MO25 protein (dee76 gene)	209	G>A	60	60	98%
	**Cpro00007487**—*Chlorella protothecoides* partial mRNA for putative extensin-like protein (dee189 gene)	134	G>del	21	60	95%
	**Cpro00009885**—*Auxenochlorella protothecoides* strain 0710 NADPH-dependent FMN reductase mRNA, partial cds	904	TGT > del	281	60	74%
	**Cpro00007215**—*Auxenochlorella protothecoides* actin gene	212 552	T>G|T A>C|A	66 461	60	60% 50%
**Week 10**	**Cpro00002508**—*Chlorella protothecoides* mRNA for putative MO25 protein (dee76 gene)	209	G>A	49	60	94%
	**Cpro00009885**—*Auxenochlorella protothecoides* strain 0710 NADPH-dependent FMN reductase	904	TGT > del or TGT	61	60	62%
	**Cpro00003782**—*Chlorella protothecoides* mRNA for putative MINE protein (dee22 gene)	126	T>G|T	58	60	53%
	**Cpro00007215**—*Auxenochlorella protothecoides* actin	212 552	T>G|T A>C|A	53 304	60	53%, 47%
	**Cpro00013040**—*Chlorella variabilis* V-type H+ ATPase subunit A (CHLNCDRAFT 56410)	20	T>G|T	24	60	46%
**>Week 14**	**Cpro00004400**—*Chlorella variabilis* ribulose-phosphate 3-epimerase mature form (CHLNCDRAFT 56033)	141 159 120 162	T>C T>C C>G G>C	41 31 43 30	60 60 60 58	100%100%98% 97%
	**Cpro00021357**—*Chlorella variabilis* 40S ribosomal protein S5 (CHLNCDRAFT 37294)	171 204 214 216	C>G C>G G>A G>C	123 201 95 94	60 60 58 58	88% 93% 87% 86%
	**Cpro00007379**—*Chlorella variabilis* ribosomal protein L19 (CHLNCDRAFT 33146)	228 261	C>G G>C	461 558	60 60	89% 87%
**>Week 22**	**Cpro00021357**—*Chlorella variabilis* 40S ribosomal protein S5 (CHLNCDRAFT 37294)	204 171 216 214	C>G C>G G>C G>A	63 40 27 27	60 60 60 60	98% 98% 96% 96%
	**Cpro00007379**—*Chlorella variabilis* ribosomal protein L19 (CHLNCDRAFT 33146)	228 261	C>G G>C	146 187	60 60	96% 95%

Note: deletion is abbreviated as ‘del’.

The SNPs/Indels were found to be scattered throughout the genome (Table 1 and S3 Table). A number of SNPs/Indels occurred in coding-regions, which were associated with different cellular processes. In the early selection stage (wk 6 and 10), SNPs/Indels were found in the coding-genes involved in cell metabolites, growth, and division. However, in the later stages of selection (>wk 14 and >22), the genomic variants were present in the coding-genes encoding a 40S ribosomal subunit and ribosomal protein (L19). Although it might have been expected that most SNPs occurring in coding regions would be synonymous, multiple SNPs were found that contributed non-synonymous changes in the ribosomal subunit encoding genes. Thus, the presence of multiple SNPs in certain ribosomal genes appear to provide functional explanation for the observed alterations in protein synthesis.

### Differential expression profiles

**Fig 5:**
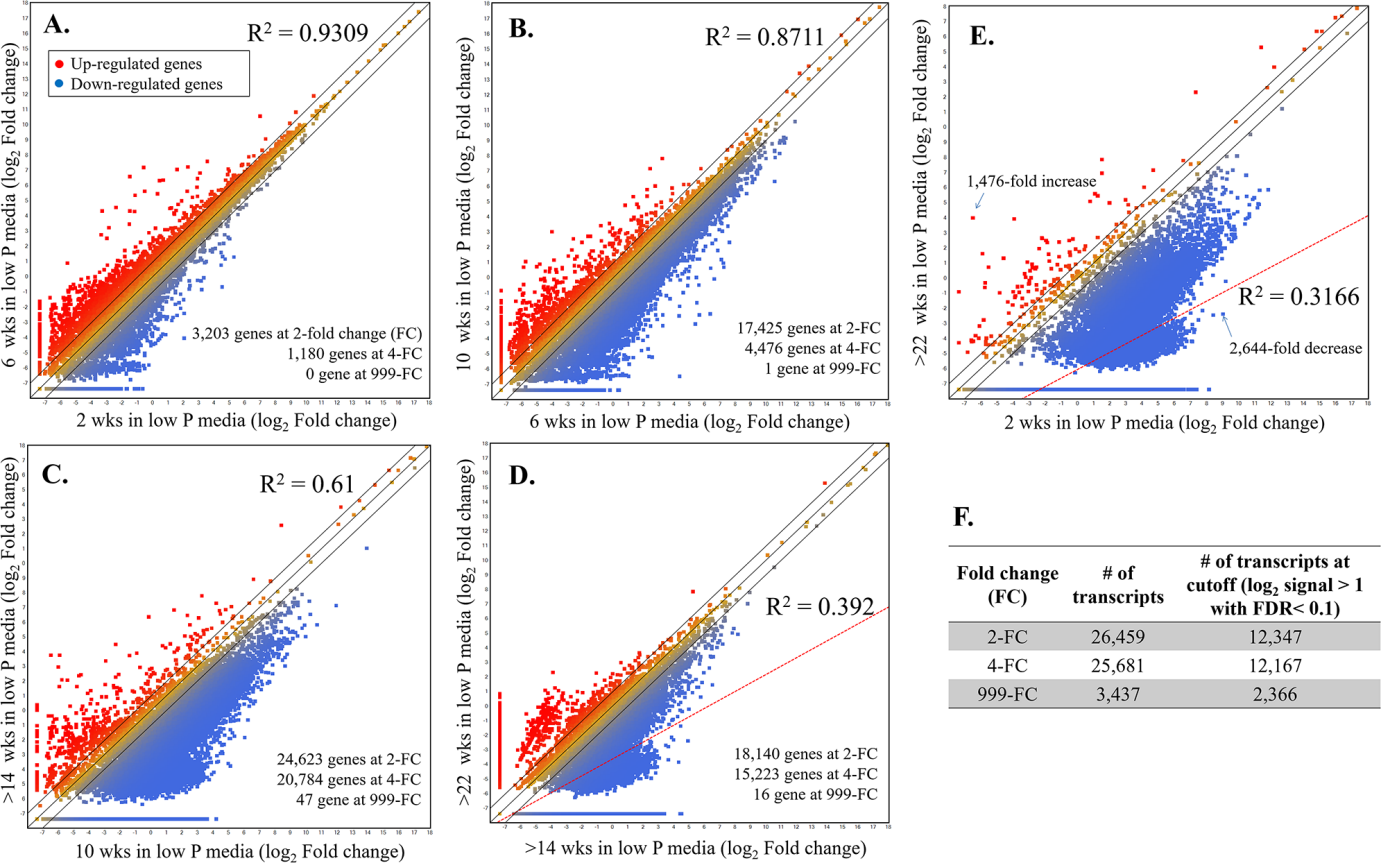
Scatter plots of gene expression expressed as log_2_ value showed pronounced variability between *A*. *protothecoides* subpopulations during low phosphate natural selection. Expression was determined from RNA-Seq reads, with 1–3 biological replicate(s), and each individual dot represents a different gene. Genes that were significantly up-regulated are shown on the X-axis in red, and down-regulated genes are shown in blue. **A**. Comparison of gene expression between wk 2 and 6 (R^2^ = 0.9309), **B**. wk 6 and 10 (R^2^ = 0.8711), **C.** wk 10 and >14 (R^2^ = 0.61), **D**. >14 and >22 wks (R^2^ = 0.392), and **E.** 2 and >22 wks (R^2^ = 0.3166). **F**. Total number of transcripts (log_2_ signal >1.00 based on the FDR multiple test correction and adjusted *p*-value < 0.1) for the wk 2 and >22 comparisons.

To examine the evidence for possible transcriptional changes associated with adaptation to low phosphate conditions, the transcriptome was determined for the low P-adapted, and the progenitor *A*. *protothecoides* strains over a 22 wk time period, and at 2 wk intervals. Fig 5 shows a scatter plot of fold change differences in transcripts occurring during low phosphate adaptation. The transcriptional comparisons indicated that the limited phosphate availability was responsible for the differential gene expression. At wk 6, down-regulation of gene expression was evident (Fig 5A), and by wk 14, significant transcriptional changes were documented (Fig 5B and 5C). These observations suggest that the *A*. *protothecoides* subpopulation approaching adaptation to the low phosphate conditions had become the predominant genotype by wk 14, indicating that the near-complete selection of low phosphate-acclimated population required as few as 14 wks to adapt to the new conditions. After this point in the selection process, however, some extent of additional transcriptional changes could be observed (Fig 5D). Based on transcript comparisons at 2 and >22 wks (Fig 5E), 12,347 transcripts were identified that exhibited differential expression, based on the cutoff, FDR adjusted *p*-value at < 0.1 for the adapted population (Fig 5F). Among these, 15 genes showed a 1.7-fold increase (or greater), and 12,332 transcripts were down-regulated by as much as 15,947-fold. In total, 398 of the down-regulated transcripts were annotatable using the BLASTn algorithm available in the NCBI-GenBank database (S1 Table).

The algal transcripts expressed at each time period, representing cells that were temporally sampled during adaptation, and the correlation value for each group, are shown in Fig 5. During the ‘early’ adaptation periods, transcripts e.g., 2 compared to 6 wks, and 6 compared to 10 wks, were linearly correlated (R^2^ = 0.9309 and R^2^ = 0.8711, respectively) (Fig 5A and 5B). However, those transcripts that were most prevalent at 10 compared to >14 wks, were minimally correlated (R^2^ = 0.61) (Fig 5C). This low value (correlation) was strongly suggestive of significant transcriptomic changes occurring between 10 and >14 wks. Once stabilized (Fig 5D), gene expression in the low P-adapted strain was altered to a lesser extent than during the adaptation period. The differential expression profiles between the wild-type *A*. *protothecoides* on wk 2 in the photobioreactor, and the selected strain after 22 wks under adaptation, exhibited the most significant changes in gene expression (R^2^ = 0.3166) (Fig 5E and 5F). The majority of transcripts were down-regulated in the adapted cultures, indicating that the low phosphate conditions exerted sufficient selection to re-program key cellular processes.

The differentially expressed genes identified in this study are assigned to different metabolic pathways (Table 2). Notably, glutamine synthetase II (Cpro00016146) expression was most significantly down-regulated, by 15,947-fold (14 at log_2_ scale), and was suggestive of the downstream reduction in glutamine, and concomitantly reduced protein biosynthesis requiring glutamine. Interestingly, three transcripts encoding chlorophyll breakdown (‘degreening’)-related proteins: the amino acid carrier (Cpro00013471), MO25 protein (Cpro00002508), MINE protein (Cpro00003782) were highly down-regulated, at > 2,400-fold, at the cutoff of the adjusted *p*-value, at <0.1. Several transcripts involved in protein synthesis were also significantly down-regulated. These transcripts were predicted: protein kinase (Cpro00016256), glucan synthase (Cpro00012778), and ribosomal subunit proteins (Cpro00024341, Cpro00023449, and Cpro00003072). Additionally, several transcripts whose gene products are predicted components of the chloroplast cytochrome b6f complex (Cpro00008847), P450 (Cpro00014746 and Cpro00008515), and plastid membrane protein (Cpro00017727) were down-regulated. Also, transcripts with predicted functions in cell development and division, including as actin (Cpro00007215) and extension-like protein (Cpro00007487), were down-regulated. Finally, a transcript encoding 26S proteasome regulatory complex protein (Cpro00021939) involved in intracellular proteolysis was significantly down-regulated in the low P-adapted strain.

**Table 2:** Selected transcripts significantly suppressed in the low P-adapted *A*. *protothecoides* strain at >22 wks.

Transcript ID	Sequence description	Fold change	Log_2_ fold change	Adjusted *P*-value
*Cpro00016146*	*A*. *protothecoides* glutamine synthetase II [GSII] mRNA, complete cds	15947.3	14.0	0.0627
*Cpro00013471*	*Chlorella protothecoides* partial mRNA for amino acid carrier [dee4 gene]	4973.2	12.3	0.0627
*Cpro00002508*	*C*. *protothecoides* mRNA for putative MO25 protein [dee76 gene]	4022.6	12.0	0.0627
*Cpro00004617*	***A*. *protothecoides* strain 0710 ATP-binding cassette transporter mRNA,** **complete cds (×3)**	2910.8	11.5	0.0627
*Cpro00003782*	*C*. *protothecoides* mRNA for putative MINE protein [dee22 gene]	2415.5	11.2	0.0627
*Cpro00016256*	*A*. *protothecoides* strain 0710 protein kinase mRNA, partial cds	2071.2	11.0	0.0627
*Cpro00009886*	***A*. *protothecoides* strain 0710 NADPH-dependent FMN reductase mRNA,** **partial cds (×2)**	1967.7	10.9	0.0627
*Cpro00008847*	*A*. *protothecoides* strain 0710 chloroplast PetM subunit of cytochrome b6f complex mRNA, partial cds	1874.0	10.9	0.0627
*Cpro00002389*	***A*. *protothecoides* strain 0710 sucrose phosphate synthase mRNA,** **complete cds (×3)**	1475.8	10.5	0.0627
*Cpro00024341*	*A*. *protothecoides* strain 0710 ribosomal protein S28e mRNA, partial cds	1413.7	10.5	0.0627
*Cpro00014746*	*A*. *protothecoides* strain 0710 cytochrome P450 like protein mRNA, complete cds	1284.5	10.3	0.0627
*Cpro00021156*	*A*. *protothecoides* culture-collection CGMCC,2578 cytosine/adenosine deaminase-like protein mRNA, partial cds	1002.2	10.0	0.0627
*Cpro00014908*	***A*. *protothecoides* strain 0710 acyl groups transferase mRNA,** **complete cds (×2)**	977.7	9.9	0.0627
*Cpro00012778*	*A*. *protothecoides* strain 0710 glucan synthase mRNA, complete cds (×3)	958.3	9.9	0.0627
*Cpro00017034*	***A*. *protothecoides* strain 0710 phenazine biosynthesis protein mRNA,** **partial cds (×2)**	948.3	9.9	0.0627
*Cpro00008515*	*A*. *protothecoides* strain 0710 cytochrome P450 like protein mRNA, complete cds	868.1	9.8	0.0627
*Cpro00017727*	*A*. *protothecoides* strain 0710 plastid integral membrane protein mRNA, complete cds	663.7	9.4	0.0627
*Cpro00010033*	***A*. *protothecoides* strain 0710 OST3/OST6 family protein mRNA,** **partial cds (×2)**	659.1	9.4	0.0627
*Cpro00021939*	***A*. *protothecoides* strain 0710 26S proteasome regulatory complex mRNA,** **partial cds (×2)**	593.9	9.2	0.0627
*Cpro00023449*	*A*. *protothecoides* strain 0710 50S ribosomal protein L27 mRNA, partial cds	550.0	9.1	0.0627
*Cpro00006025*	*A*. *protothecoides* culture-collection CGMCC,2578 4-alpha-glucanotransferase mRNA, partial cds	448.7	8.8	0.0627
*Cpro00000168*	*A*. *protothecoides* strain 0710 PHD-type transcription factor mRNA, complete cds	399.1	8.6	0.0627
*Cpro00003072*	*A*. *protothecoides* strain 0710 50S ribosomal protein L3 mRNA, partial cds	139.8	7.1	0.0627
*Cpro00007215*	***A*. *protothecoides* actin gene, complete cds (×2)**	121.6	6.9	0.0627
*Cpro00014133*	*A*. *protothecoides* strain 0710 glycine rich protein mRNA, complete cds	56.8	5.8	0.0627
*Cpro00015751*	*C*. *variabilis* argininosuccinate lyase [CHLNCDRAFT 26929] mRNA, complete cds	26.2	4.7	0.0627
*Cpro00014004*	*A*. *protothecoides* strain 0710 malate dehydrogenase mRNA, partial cds	17.3	4.1	0.0627
*Cpro00007487*	*C*. *protothecoides* partial mRNA for putative extensin-like protein [dee189 gene]	3.6	1.8	0.0627
*Cpro00013040*	*C*. *variabilis* V-type H+ ATPase subunit A [CHLNCDRAFT 56410] mRNA, complete cds	2.6	1.4	0.0627

The sequence descriptions in bold indicate different isoforms. The fold change was rescaled as a log_2_ value with the FDR multiple test correction, and adjusted *p*-value < 0.1.

Although it is not possible to determine the specific cellular mechanisms that were affected without assessing overall global patterns in detail, these experimental results provide a snapshot of some predominant transcripts whose expression levels were altered under limited phosphate conditions, and indicate with reasonable certainty that about 99% of the transcripts considered in the templated assemblies were down-regulated to at least some extent. Thus, the results showed that the majority of cellular processes were attenuated, while at the same time, the algal cells were able to maintain the capacity for expression of genes vital for cellular metabolism.

**Fig 6:**
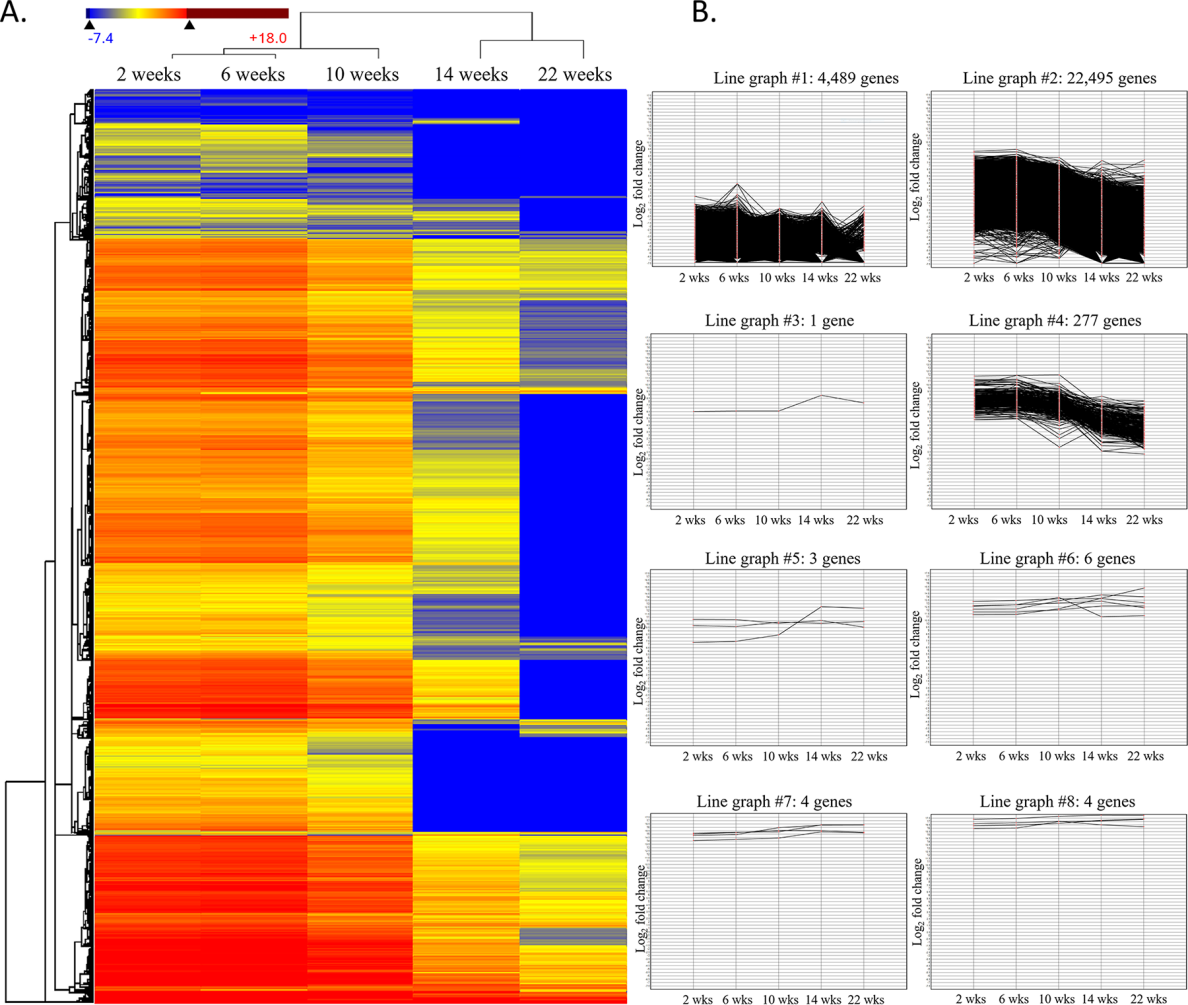
**A**. A cluster heat map displaying the change of gene expression. Each column represented a different selection stage and the each low represented the level of transcription expressed in the color scheme. In total, 27,279 transcripts were compared across the selection process. The transcript levels observed at wk 2 were considered as the basal transcriptional level for comparison with gene expression levels observed at 6, 10, >14, and >22 wks. In general, transcripts were hierachically clustered, based on analysis using the Centroid method and Euclidean distance metric. **B.** The line graphs shows different gene expression patterns among the 27,279 transcripts, indicative of low phosphate selection. Among the 27,279 transcripts, >99.9% transcripts showed a gradual decrease in gene expression (line graph #1,#2, and #4), whereas, increased expression was observed for only <0.1% of transcripts (line graph #7 and #8).

To determine the extent of adaptive evolution of the *A*. *protothecoides* low P-adapted strain, gene expression patterns over the course of the adaptation process were analyzed. The results were visualized by plotting a cluster heat map (Fig 6A) and line graphs (Fig 6B). Transcripts from wk 2 were designated as the ‘basal gene expression level’, for comparison with transcriptional levels for the selected time points of wk 6, 10, >14, and >22. Results indicated that the majority of transcripts, at 99%, were down-regulated, over time (line graph #1, #2, and #4), and that only a few genes, at <0.1%, showed increased (line graph #7 and #8) or mixed (up and down) (line graph #3, #5, and #6) expression patterns. The most signicant reduction occurred after wk 14, while another decline occurred near the end of the adaptation period, at wk 22.

### Metabolic pathways analysis

The down-regulated transcripts were associated with a number of metabolic pathways, including purine and pyrimindine-, aminoacyl-tRNA-, amino acid-, starch-, and sucrose-metabolism. These gene products are essential for cell wall biosynthesis, carbon fixation, and lipid biosynthesis. This pattern of differential gene expression indicated that the limiting phosphate resouces negatively affected certain cellular processes. Enzymes such as those involved in nucleotide, aminoacyl-tRNA and amino acid biosynthesis that are essential for cell growth and devleopment were significantly down-regulated, as well as enzymes involved in cell wall membrane biosynthesis, e.g., N-glycan, lipopolysaccharides, glycerophospholipid and peptidoglycan. In addition, transcripts involved in secondary metabolite biosynthesis, specifically, carotenoid and fatty acid, were significantly down-regulated. Unexpectedly, down-regulation of the expression of the latter genes did not result in reduced biomass, as might have been expected, however, it is not possible to distinguish between direct and indirect affects of the altered expression patterns.

**Fig 7:**
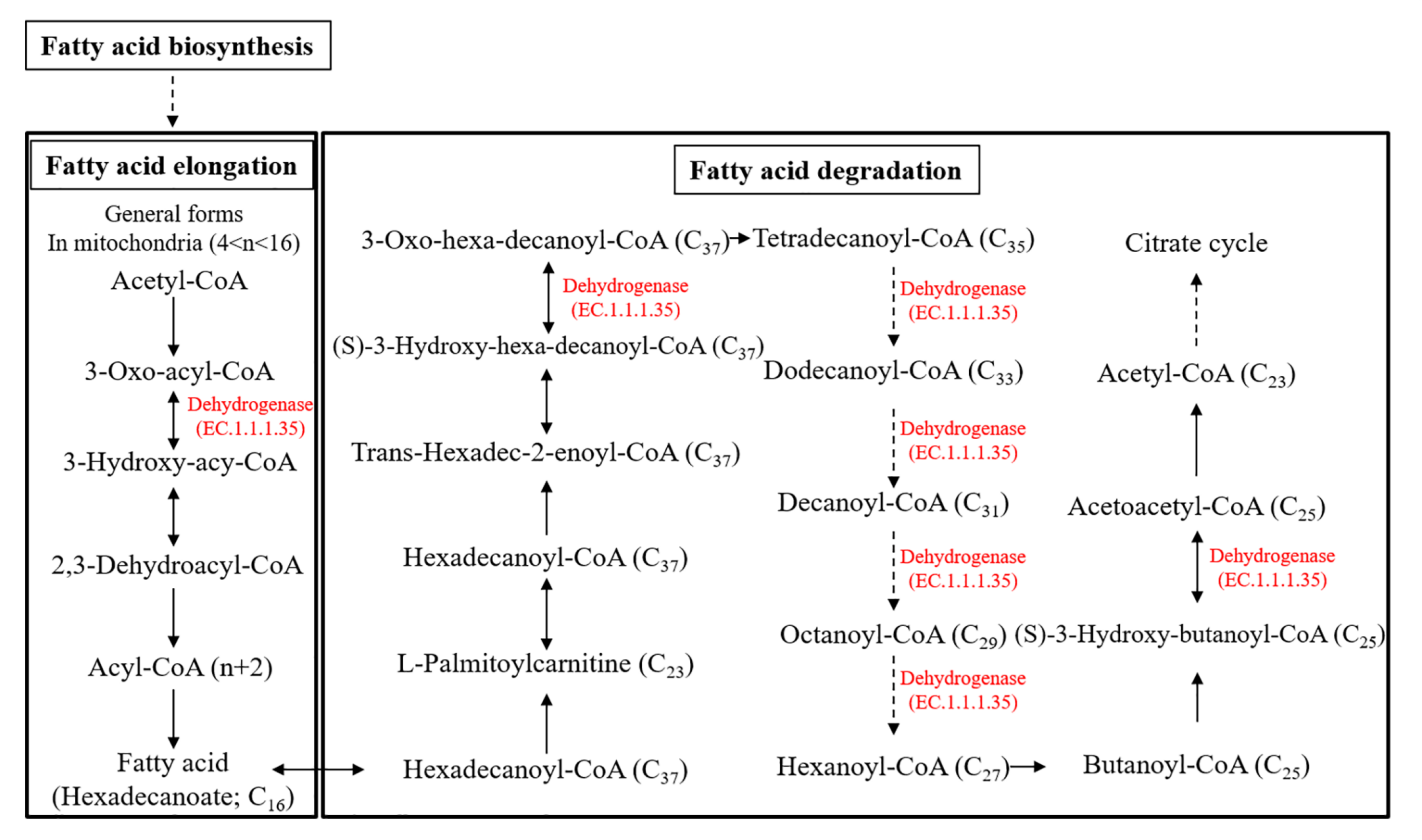
The fatty acid elongation and degradation KEGG pathway showing the role of dehydrogenase (E.C. 1.1.1.35; 5374-fold decrease) in fatty acid elongation and degradation.

In this study, lipid metabolism of the low P-adapted strain was investigated. Fatty acid elongation and degradation are key steps that determine the length of the carbon chains. Notably, dehydorgenase E.C.1.1.1.35 was significantly down-regulated, by 5,374-fold, in the low P-adapted strain. The enzmyes found in both fatty acid elongation and degradation pathways are essential to determining the length of the carbon backbone (Fig 7). Therefore, down-regulation of dehydrogenase would be expected to influence fatty acid and lipid composition.

### Calorific value analyses of fatty acids

**Fig 8:**
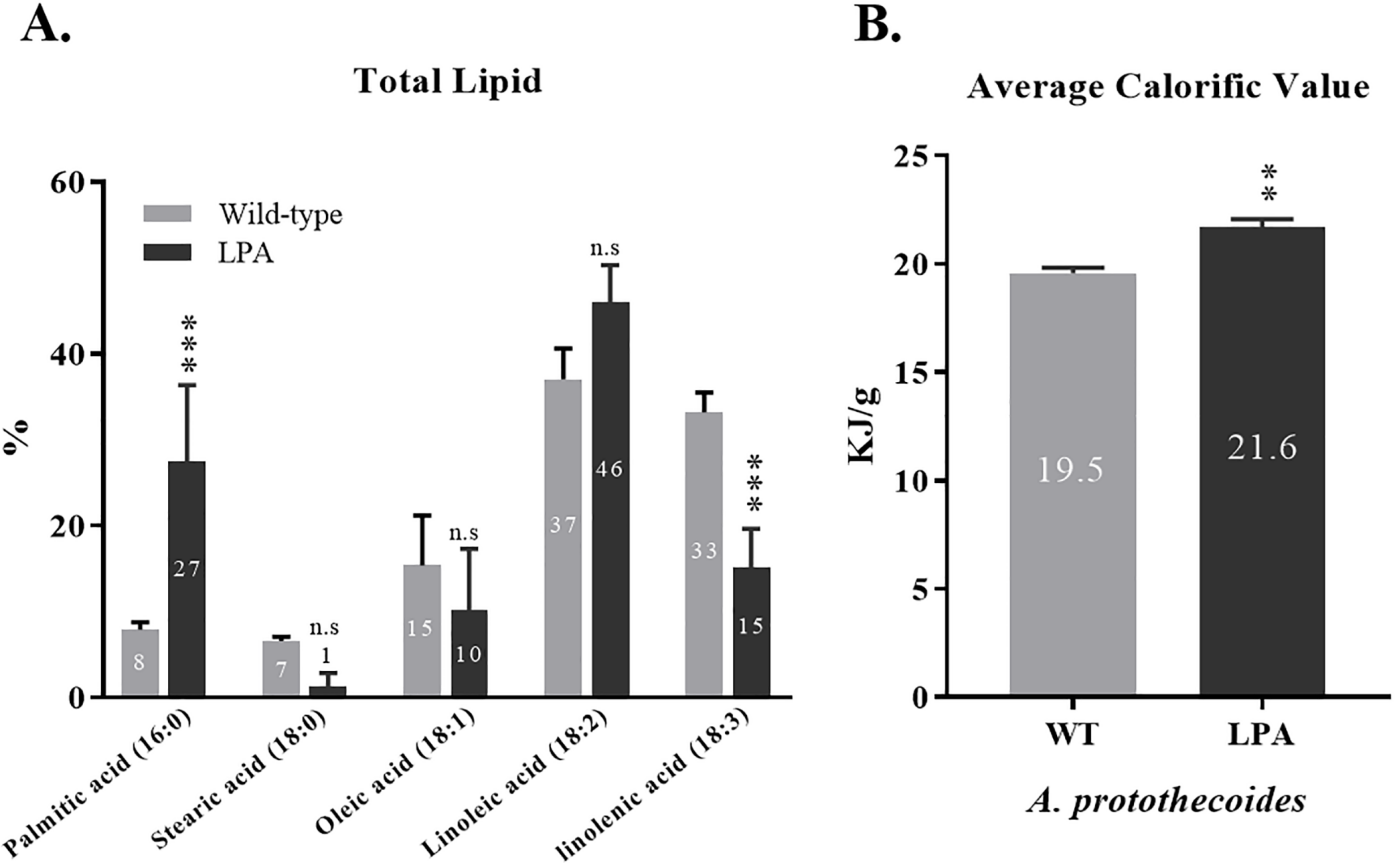
Fatty acid and calorific value analyses of wild-type and low P-adapted strain. **A**. Compositional changes in fatty acids based on GC-FID analysis in wild-type and low P-adapted *A*. *protothecoides* (one-way ANOVA, Tukey’s multiple comparisons test, n = 3, P<0.0001). **B**. Increased average calorific values for the low P-adapted strain (t-test, n = 3, P = 0.0013).

To determine if fatty acid composition was altered in the low P-adapted strain, Gas Chromatography with Flame Ionization Detector (GC-FID) and Liquid Chromatography equipped with Mass Spectrometry (LC-MS) analysis were carried out. Results indicated that palmitic acid (16:0) was increased by 3.3-fold for the low P-adapted strain, while linolenic acid (18:3) was decreased by 2.2-fold (Fig 8A). The contribution of stearic acid (18:0), oleic acid (18:1), and linoleic acid (18:2) was unchanged.

The calorific content analysis revealed that the low P-adapted *A*. *protothecoides* strain was 11% greater than the wild type, at 21.6 kJ/g for the low P-adapted strain compared to 19.5 kJ/g for the wild-type strain (Fig 8B), indicating that lipid content and caloric value were correlated, observations that are corroborated by the fatty acids profiles. Specifically, the wild-type and low P-adapted strains differed, with the predominant change being 3-fold greater production of palmitic acid, indicative of the increased calorific value observed for the low P-adapted, compared to the wild-type strain.

### LC-MS lipid composition

**Fig 9:**
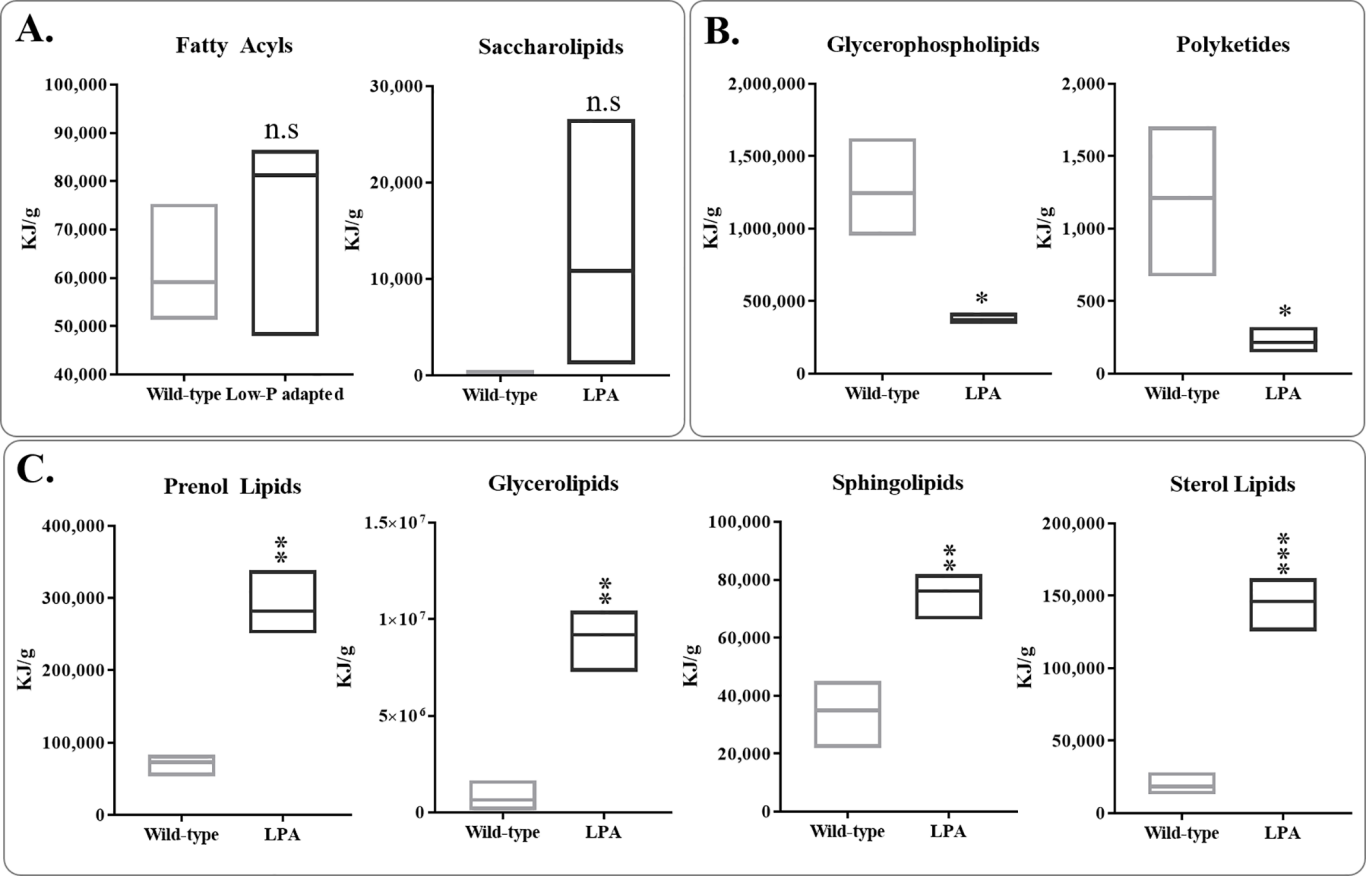
Lipid composition of wild-type and low P-adapted strain by LC-MS analysis. **A**. Fatty acyls and saccharolipids were produced at similar levels by both strains (t-test, n = 3, P>0.05). **B**. Glycerophospholipid and polyketide accumulation was less in the low P-adapted compared to wild type strain (t-test, n = 3, P<0.05). **C**. Prenol lipids, glycerolipids, sphingolipids, and sterol lipids were increased in the low P-adapted strain (t-test, n = 3, P<0.05).

Liquid chromatography-mass spectrometry (LC-MS) was carried out to detect potential changes in lipid composition between the wild-type and low P-adapted strain. All eight lipid categories: fatty acyls, glycerolipids, glycerophospholipids, sphingolipids, sterol lipids, prenol lipids, saccharolipids, and polyketides, were analyzed. Among them, the production of two classes of lipids, fatty acyls and saccharolipids, were not altered to any noticeable extent in the wild-type or low P-adapted strain (Fig 9A). The production of glycerophospholipids and polyketides was significantly lowered (Fig 9B), while prenol lipids, glycerolipids, sphingolipids, and sterol lipids were significantly increased in the low P-adapted strain (Fig 9C).

## Discussion

Phosphate is an essential nutrient required for the synthesis of nucleic acids, amino acids, catalytic enzymes, and other molecules necessary for maintaining cell growth and development. Large-scale algal production characteristically involves the addition of 25 mg ammonium dihydrogen phosphate (NH_4_H_2_PO_4_) per liter of starting culture, with increasingly greater amounts added during scale-up. For algal production to continue to be economical, the cost of phosphate, one of the most expensive nutrients, must be reduced.

In this project, we used an adaptive evolution approach to select for a low P-adapted strain of *A*. *protothecoides*. A wild-type culture was grown for over 10 generations in proteose media with a 100× lower phosphate and 5× lower peptone compared to traditional media. Once the adaptation was underway, evidence of selection occurred in approximately 45–50 days, and stable adaptation occurred after 95 days. The follow up experiment demonstrated that compared to the parental population, the low P-adapted *A*. *protothecoides* produced ~ 25% higher biomass based on OD_600_ measurements, corresponding to a biomass AFDW increase of 30% (up to 0.293 g/L AFDW), when both strains were grown in the low phosphate media (Fig 1D), while sustaining the production of equal amounts of total lipids (~10% lipid yield). In a control experiment the wild-type culture was grown with regular media in the chemostat. This culture showed stable growth after 8 days with no signs of adaptation for 20 days (Fig 1C), which is consistent with the absence of detectable adaptation without selective pressure.

The RNA-Seq analysis showed that the low P-adapted strain was distinguishable from the wild-type strain at both the genomic and molecular level. The RNA-Seq profiling showed that growth under phosphate-limited conditions resulted in the reshaping of gene expression patterns that suggested the utilization of less phosphate was made possible by greatly reducing non-essential cellular activities that required phosphate.

A comparison of the genomic and RNA-Seq sequences of the wild-type parent and low P-adapted *A*. *protothecoides* indicated the presence of SNPs and Indels indicative of single nucleotide or sequence regions that were unique to each strain. Further, synonymous substitutions at SNPs and Indels were mutations distributed primarily in noncoding regions of the algal genome, features that were interpreted as naturally-occurring variation in response to selection. Whether the adapted strain, which shared significant sequence identity with the wild-type strain, is a new genetic variant, or more likely, represents a minor (or non-predominant) sub-population among others that were integral to the parental population, is not yet clear.

The RNA-Seq heat map revealed gradual changes in gene expression over time, coincident with adaptation in response to the low phosphate conditions (Fig 6A). The relatively slow changes in temporal expression patterns indicated that the low phosphate environment was not lethal, and that it provided ample selection against sectors of the population for which the conditions were suboptimal. Also, *A*. *protothecoides* responded to phosphate deficiency by down-regulating the expression of glutamine synthetase II and degreening (chlorophyll breakdown)-related transcripts (Table 2). And, the resultant tuning of transcriptional expression patterns was indicative of responses to environmental change that perhaps would be considered more efficient than would modifying core genomic information.

Among multicellular organisms, there are several possible options for regulating gene expression. In general, the expression of transcripts is largely dependent upon a suite of regulatory elements e.g., promoter, transcriptional factors. Small RNA molecules, or microRNAs (miRNA) of 21–23 nt in length are known to be powerful mediators of gene expression and function either by degrading a target transcript or by repressing translation. The target specificity of miRNA enables precise regulation of gene expression in different tissues, organs, and growth stages. The results of RNA-Seq analysis revealed that the low P-adapted *A*. *protothecoides* expressed approximately 436 miRNAs, based on a BLASTn search of the miRNA *Arabidopsis thaliana* database, representing genes involved in cellular mechanisms feasibly associable with phosphate-deficient condition.

In summary, for the low P-adapted algal strain, >99% of transcripts that are involved in amino acid/protein synthesis, cytochrome b6f complex, cell development and division, and intracellular proteolysis were down-regulated at cutoff value of adjusted *p*-value <0.1 with 99% confidence level (Table 2 and S1 Table), a pattern that could be interpreted by a negative effect on protein synthesis during early stages of selection (adaptation). Such a dramatic shift in gene expression might have been expected to negatively affect cell growth, at least in part due to the perturbed synthesis of the cell membrane, which is composed largely of phospholipids. Early in the selection process, no transcripts were significantly changed above cutoff of the adjusted *p*-value <0.1. The gene expression began to alter at wk 6. When transcripts collected at wk 6 were compared to wk 10, a total of 1,470 transcripts were differentially expressed. Most of these transcripts were annotated as a hypothetical protein, except for one that encoded a degreening-related protein (*dee25*) that was found in chlorophyll breakdown process of *A*. *protothecoides* grown in nitrogen-free bleaching medium [24], at a 6.4-fold decrease. The *dee25* gene appeared to be down-regulated during the early stage of low phosphate selection. A comparison of gene expression at wk 10 and at >14 wks, a total of 7,322 transcripts appeared to be differentially expressed above the cutoff of the adjusted *p*-value <0.1. These transcripts were involved in different cellular processes, including amino acid/protein synthesis (amino acid carrier, glucan synthase, 50 ribosomal protein), oxidoreduction (NADPH-dependent FMN reductase, P450-like protein), cell development and division (extension-like protein, MINE), nucleotide modification (cytosine/adenosine deaminase-like protein), proteolytic activity (proteasome regulatory complex), and others (ammonium transporter gene, ATP-binding cassette transporter, sucrose phosphate synthase, malate dehydrogenase, ATPase subunit A, and acyl groups transferase). Interestingly, two copies of transcripts encoding phenazine biosynthesis protein were significantly down-regulated by 948-fold which has shown antimicrobial activities [25]. The same group of transcripts were also appeared in the low P-adapted strain at wk >22 (Table 1 and S1 Table). The increase number of transcript and fold-change between the wk 10 and wk >14 suggest that tremendous transcriptional expression changes have occurred in this adaptation period. When at wk >14 and >22 were compared, a total of 3,238 transcripts were identified but none were observed above the cutoff of adjusted *p*-value < 0.1. It is important to note that the same group of transcripts found in the wk 10 and >14 was identified in the wk >14 and >22 comparison. The same group of genes appeared to be further down-regulated, although the FDR cutoff value did not meet the cutoff criteria (adjusted p-value<0.1) (S2 Table).

Based on LC-MS analysis, the composition of palmitic (16:0) and linolenic acid (18:3) in the wild-type *A*. *protothecoides* strain was 8% and 33% per dry weight, respectively. However, the compositional shift occurred as the palmitic acids increased up to 3.3-fold (27% per dry weight) while linolenic acids decreased by 2.2-fold (15% per dry weight) in the low P-adapted strain. The two strains produced nearly the same amount of total lipids (~10% / dry weight), however, the low P-adapted strain produced more saturated fatty acids. With respect to energy yield, the low P-adapted strain produced and stored fatty acids having a higher caloric value than the wild-type strain, by storing fatty acides as saturated palmitic acid (16:0) instead of unsaturated linolenic acid (18:3). In general, compared to unsaturated fatty acides, saturated forms store more energy in the additional extra hydrogen bonds. This hypothesis is consistent with the observed reduced gene expression of dehydrogenase (E.C.1.1.1.35), which is involved in fatty acid carbon elongation and degration, thereby explaining the 3.3% increase in palmitic and a concommitant 2.2% decrease in linolenic acids (Fig 9).

## Conclusions

Over 10 generations, the low P-adapted *A*. *protothecoides* strain became the predominant population in the culture, with the capacity to produce more biomass than the wild type population, without compromising lipid yield. Evidence provided herein strongly suggests that the selected *A*. *protothecoides* strain ‘prioritized’ the use of the limited phosphate for use in vital biological processes, over cell growth and development. The transcript sequences of the low P-adapted strain selected by adaptive evolution was nearly identical with respect to genome sequence, to the *A*. *protothecoides* genotype used as the starting culture for the initial inoculation of the chemostat media. The improved performance (growth) of the low P-adapted isolate appears possible by the reshaping of overall gene expression to facilitate the lowered requirement for phosphate, and did so by greatly reducing lesser and/or non-essential cellular activities. Indeed, the low phosphate *A*. *protothecoides* population showed a 3% increase in biomass production, and 1.1× higher calorific value, compared to its wild-type parent population. This low P-adapted isolate of *A*. *protothecoides* therefore has great potential for use in the commercial production of biodiesel fuel, at a lowered cost with respect to overall inputs of phosphate, particularly during scale-up, than its wild type algal counterparts. In addition, the cost of culture maintenance is expected to be less than that of the wild-type parent, and potentially, can be grown with less attention to quality control to abate pond invasion by competitors and other pest organisms seeking substrate phosphate. Finally, the enhanced performance of microalgae selected under natural conditions, compared to genetically-engineered genotypes, are free of environmental and related-health concerns, particularly in outdoor production systems.

## Supporting information

S1 Table Selected transcripts that were significantly changed in the low phosphate adapted *A*. *protothecoides*. (XLSX)

S2 Table Transcriptional changes during low phosphate adaptation. (XLSX)

S3 Table Selected list of SNPs/Indels identified from each selection process under low phosphate condition. (XLSX)
